# Do Overweight People Have Worse Cognitive Flexibility? Cues-Triggered Food Craving May Have a Greater Impact

**DOI:** 10.3390/nu14020240

**Published:** 2022-01-06

**Authors:** Shiqing Song, Qingqing Li, Yan Jiang, Yong Liu, Aidi Xu, Xinyuan Liu, Hong Chen

**Affiliations:** 1 Faculty of Psychology, Southwest University, Chongqing 400715, China; song_19921014@163.com (S.S.); 17784729185@163.com (Q.L.); jiangyan_12@126.com (Y.J.); liuy0768@swu.edu.cn (Y.L.); liuxiny2019@163.com (X.L.); 2 Key Laboratory of Cognition and Personality (SWU), Ministry of Education, Chongqing 400715, China; 3 Faculty of Health, Department of Psychology, York University, Toronto, ON M3J 1P3, Canada; ivyx1124@gmail.com

**Keywords:** overweight, cognitive flexibility, proactive control, reactive control, food cravings

## Abstract

Background: Overweight people have been revealed to have poor cognitive flexibility. Cognitive flexibility reflects proactive and reactive control abilities. However, the impairment had not been explicitly positioned at the cognitive stage. Therefore, this study provides increased support for impairment of cognitive flexibility due to overweight. Method: The study included 34 overweight and 35 normal-weight participants. They were required to complete the food and flower target AX-continuous performance test (AX–CPT), including the resting-state fMRI and cue-triggered food craving subscales. We compared the performance difference between the two tasks. Furthermore, we investigated whether the cue-triggered food cravings and the corresponding brain regions mediated the effect of overweight on the two control mechanisms. Result: Significant differences were found only in the food target AX-CPT task, where overweight participants performed worse. Cue-triggered food cravings mediated this relationship. Additionally, we found that the brain regions associated with cue-triggered food cravings (bilateral SFG) can completely mediate the relationship between BMI and the z-value of the fat mass index and sensitivity to proactive control. Conclusion: In the food target task, overweight participants performed worse in both control mechanisms. Moreover, we also revealed the potential mechanism by which being overweight might affect the two control mechanisms through cue-triggered food cravings.

## 1. Introduction

Overweight and obesity are becoming major public health concerns. The prevalence of obesity is rising rapidly worldwide, especially among adolescents and young adults. Moreover, evidence suggests that overweight, obesity, and excessive consumption of high-energy foods are harmful to brain development and have a non-negligible destructive effect on executive function [1,2,3,4,5,6]. In food-related tasks, people who are overweight and obese have been found to perform worse in cognitive control, but are more sensitive to food cues [7,8]. Food makes it more difficult to control impulsive and flexible reactions because high-energy foods represent high rewards.

Previous studies have provided behavioral and neurological evidence that overweight people have poorer cognitive flexibility [3,9], and the effect was confirmed by different paradigms [10,11]. Neurological evidence for overweight individuals in switching tasks suggests that cognitive flexibility is associated with the activation of the dorsolateral prefrontal cortex, ventrolateral prefrontal cortex, inferior frontal gyrus, superior temporal gyrus, auxiliary motor areas, and parietal gyrus [12,13,14,15,16]. The neural mechanisms of cognitive flexibility that are affected by overweight include high-level executive control brain regions and the perception and emotional processing cortex.

Cognitive flexibility enables humans to rapidly change cognition and reaction according to changes in the environment and task goals [17]. To deal with complex tasks, individuals adjust the allocation of attention and cognitive resources, including top-down initiative control and bottom-up passive regulation [18]. Based on this view, researchers have proposed a dual mechanism to clarify the cognitive control account that affects the cognitive and transfer processes that affect cognitive flexibility [18]. The theory supports that switching includes two components: proactive control and reactive control. Proactive control is a cue-driven control process [18] that requires the participation of task-set reconfiguration. Individuals need to suppress previous response strategies and establish new coping rules. This process relies on the activation of the lateral prefrontal cortex (PFC [6]). Reactive control is a post-correction process that reflects the reactions to new response strategies after monitoring conflicts [18]. This process is considered to be the transient activation of the prefrontal lobe and other brain regions such as the anterior cingulate cortex (ACC [19]). These two control processes are independent and separate. They involve cognitive control and need attention to allocation and establish appropriate stimulus–response connections [20]. Importantly, cognitive flexibility not only depends on proactive control through cues but also the ability to adjust responses to stimuli. In this study, we used the AX-continuous performance test (AX-CPT) to explore the flexibility of cognitive conversion. Based on the signal detection theory, a *d’* index was calculated to analyze the proactive and reactive control abilities of participants [18,21].

Previous studies have indicated that compared with general stimuli, food-related stimuli induce higher motivation or emotional involvement in overweight people, which might be an important factor in weight gain [22]. Similar results were found in the case of restrained eaters. In the food-specific switching task, the P3 amplitude increased significantly, implying that more cognitive resources were needed to suppress food-specific stimuli [23]. These results suggest that food has a substantial reward value for certain groups of people (overweight and obese people and restrained eaters). Additionally, rewards may induce bias in reactive control [19]. Therefore, it is necessary to compare the performance of overweight people in food reward stimulation and non-food stimulation tasks and explore whether key neural nodes belong to the reward system.

For a long time, brain regions related to the reward system have been considered important in regulating body weight and eating behavior. These regions are related to emotion, memory, sensory processing, motor function, and cognitive control. Brain-imaging evidence has shown that overweight adolescents respond to sweetened beverages through addiction pathways. This leads to hyperactivity in reward-related regions, including the putamen, caudate nucleus, orbitofrontal cortex (OFC), prefrontal cortex (PFC), anterior insular cortex, anterior cingulate cortex, parahippocampal cortex, thalamus, and posterior cingulate cortex [24]. These brain regions are involved in pleasure and reward processing, food cravings, and monitor reward processing. Clinical studies on female obesity have found that the ventral striatum, caudate nucleus, and OFC are the key nodes related to rewards. They enhance the visual processing of information and further participate in cognitive, attention, and sensorimotor processing [25,26]. Moreover, previous studies have shown similarities between drug addiction and food addiction in individuals with overweight and obesity [27,28]. These reward-related brain regions exhibit abnormal activation during food- or addiction-related stimuli [29,30]. Studies have shown that marijuana can increase activity in many brain regions, especially reward pathways, including the bilateral amygdala, hippocampus, and OFC [25]. Increasing evidence suggests that changes in reward-related regional activity may lead to food addiction or hedonic eating, which is also associated with weight gain [27]. Therefore, comparing the similarities and differences in cognitive conversion flexibility between food-stimulated and non-food-stimulated tasks among overweight people is necessary.

Cognitive flexibility is an executive function that is composed of complex cognitive processes. Although prior studies have explored the effect of overweight and obesity on cognitive flexibility [31], few studies have examined whether this effect occurs at different cognitive stages. Separate psychological or neural mechanisms may cause this difference. Therefore, this study investigated whether there is a difference in cognitive flexibility in cognitive processing tasks between overweight and normal-weight participants. Specifically, we tested the difference between reactive and proactive control among normal-weight and overweight individuals. Considering that food has special reward signals for obese and overweight individuals [32], researchers compared the behaviors of the two groups in food-specific tasks and general switching tasks and discussed the relationship between food cravings. It is crucial to determine the cognitive mechanisms of overweight/obesity that affect cognitive flexibility. Furthermore, by observing the negative impacts of overweight at different cognitive stages on cognitive flexibility, this study can provide a theoretical basis for further intervention research. Based on previous evidence, we propose the following hypotheses:

**Hypothesis** **1.** *Compared to the normal-weight group, overweight participants perform worse in the AX-CPT and are less sensitive to cue and target information, especially in the food target task.*

**Hypothesis** **2.** *The relationship between overweight, reactive control, and proactive control is mediated through cue-triggered food cravings.*

**Hypothesis** **3.** *The reward brain area related to cue-triggered food cravings might be the neural mechanism for worse reactive and proactive control in the overweight group.*

## 2. Method

This study was ethically approved by the Southwest University Human Research Ethics Committee (No. H20067). All participants signed the ethical committee-approved informed consent form before scans.

### 2.1. Participants

Participants were recruited through campus advertisements. A total of 69 college students (30 males, 39 females), aged 18–26, with a BMI range from 18.5 (kg/m^2^) to 29.9 (kg/m^2^), were included in the study. According to the BMI cut-off points proposed by the World Health Organization (WHO) [33,34], 34 overweight participants (25.0–29.9 kg/m^2^) and 35 normal-weight participants (18.5–24.9 kg/m^2^) took part in the study. They all completed the collection of body mass index (BMI), fat mass index (FMI), skeletal muscle mass (SMM), and AX-continuous performance test, but due to the lack of food-craving scale and fMRI data, one participant was excluded from subsequent analysis. The data were collected from October 2020 to November 2020. The measurement time was set in the morning. Before the weight and height measurements, the participants were instructed not to eat or drink alcohol or coffee within eight hours. All participants were right-handed, with normal or corrected-to-normal vision, had no history of eating disorders, and did not engage in long-term physical activity, sports, or fitness training.

Additionally, we used G*Power analyses to ensure that the sample sizes were adequate to detect the effects of interest. The post-hoc power analysis showed that with 68 participants, the power (1−β) was 0.82.

### 2.2. Procedure

Well-trained research assistants obtained resting-state fMRI scans and behavioral measurements. Subjects self-reported their sex and age, and used a visual analog scale (VAS) of 0–100 (0 represents not hungry at all, 100 represents very hungry) to assess the degree of hunger.

#### 2.2.1. Body Mass Index, the z Score of Fat Mass Index, Skeletal Muscle Mass

The WHO BMI classifications of overweight and obesity are intended for international use. Measurements of height were accurate to 0.1 cm, and those of weight were accurate to 0.1 kg. BMI was calculated using the standard formula weight (in kilograms) divided by height (meters) squared (BMI = Weight (kg)/Height^2^ (m^2^)). The FMI (kg/m^2^) and SMM (kg/m^2^) were measured using the SECA medical human body component analyzer. Previous studies have suggested that the FMI and SMM are essential indicators to distinguish people with obesity from those with normal weight. FMI were converted to age, sex, and race-specific z-scores, based on reference ranges generated in the National Health and Nutrition Examination Survey [35,36]. This study also compared the z-score of FMI, BMI, and SMM between the two groups in order to provide high accuracy in the grouping. Both BMI and FMI (z-score) were included as indicators of overweight in this study.

#### 2.2.2. Cue-Triggered Food Cravings

Cue-triggered food cravings (CTFC) were assessed using the ”cues that may trigger food cravings” subscale, characteristic of the Food Cravings Questionnaire—Trait. There are 4 items (e.g., It is hard for me to resist the temptation to eat appetizing foods that are in my reach) in this subscale. Respondents expressed the frequency with which each comment was correct for them on a 6-point scale: never (or not applicable), rarely, sometimes, often, usually, or always.

#### 2.2.3. AX-Continuous Performance Test (AX-CPT)

**Figure 1:**
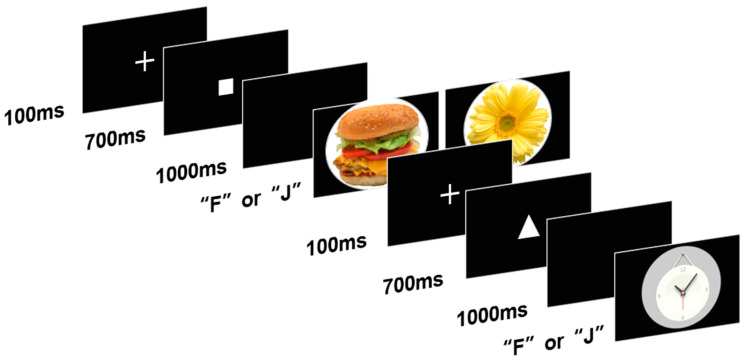
Schematic representation and time course of the cues and the stimuli adopted in the AX-CPT.

The AX-CPT paradigm was used to evaluate reactive and proactive control. The tasks and experimental conditions are shown in Figure 1. The paradigm comprised two types of tasks: a food-target task and a flower-target task to investigate whether the two groups performed differently on food and non-food tasks.

In the task, each trial began with a cue, which was either a “square” or a “triangle.” After a stimulus interval of 1000 ms, the target appeared. In this task, two types of pictures (foods or neutral objects in the food-target task, and flowers or neutral objects in the flower-target task) were randomly presented in the center of the screen. The paradigm included four types of trials: 70% consisted of a “square” followed by a neutral object picture (AX trials), 10% consisted of a “square” followed by a food (flower) picture (AY trials), 10% consisted of a “triangle” followed by a neutral object picture (BX trials), and 10% consisted of a “triangle” followed by a food (flower) picture (BY trials). Whenever participants observed an AX trial, they were required to press “F” and when they observed other trials they had to press “J” as quickly as possible in both cases. Tasks automatically entered the subsequent trial after the participants made a choice.

We recorded the error rates (ERs) and response times (RTs; correct responses only) separately for the four trial types (AX, AY, BX, and BY). The error rate and response time of the AY trial reflect the subject’s reactive control, and BX reflects the proactive control [21,37]. Based on the signal detection theory [38], we calculated the discriminant index to represent their proactive and reactive control abilities. A higher ER and RT indicate a lower reactive control and proactive control ability. The *d’*-target index was calculated by computing the discriminative index from hit AX trials and false alarms in AY trials (*d’*-target index = Z(AX-hit) − Z(AY-false alarm)). The index reflects the sensitivity to nonpersistent stimulation (food or flower). The higher the *d’*-target index score, the higher is the sensitivity to the response to the stimulus. The *d’*-context index was calculated by computing the hit AX trials minus false alarms in the BX trials (*d’*-context index = Z(AX-hit) − Z(BX-false alarm)). The index reflects the sensitivity to the cue (square or triangle). A higher *d’*-context index score indicates higher sensitivity to distinguishing the cues.

### 2.3. fMRI Data Acquisition and Processing

Each subject underwent an eight-minute resting-state fMRI (rs-fMRI) scan by using a 3T Trio scanner (Siemens Medical, Erlangen, Germany) at the Brain Imaging Center. For each subject, 240 contiguous volumes were acquired with a gradient echo-planar imaging sequence. The scanning parameters were as follows: repetition time = 2000 ms, echo time = 30 ms, slices = 62, slice thickness = 2 mm, flip angle = 90°, field of view read = 224 × 224 mm^2^, resolution matrix = 112 × 112, voxel size = 2 × 2 × 2 mm^3^, and phase encoding direction = PC >> AC. In particular, all the participants wore foam pads and earplugs to reduce head motion and scanning noise and were instructed to keep still and close their eyes, avoid falling asleep, and not think about anything during the MRI scan. For all participants, none of them had head motion between volumes in any direction >3 mm or rotation in any axis > 3 during scanning, and the mean framewise displacement (FD) values did not exceed 0.50.

The resting-state fMRI images were pre-processed using the Data Processing & Analysis for Brain Imaging (DPABI, v4.5, www.rfmri.org/dpabi, accessed on 15 September 2021) and SPM12 toolkits (www.fil.ion.ucl.ac.uk/spm/software/spm12, accessed on 15 September 2021). In order to keep the fMRI signal stability, the first 10 images were excluded. The remaining images were corrected for temporal shifts between slices and head motion and realigned to the middle volume. Next, the images were spatially normalized into the standard stereotactic Montreal Neurological Institute (MNI) space, with a resolution voxel size of 3 × 3 × 3 mm^3^. Then, they were spatially smoothed with a 4 mm full width at half maximum (FWHM) of an isotropic Gaussian kernel, and linear trends were removed afterward. After this pre-processing, we used a multiple linear regression analysis to control the effects of potential physiological artifacts, including white matter, cerebrospinal fluid, global signals, and head motion parameters (Friston 24 parameter mode).

### 2.4. fALFF-Behavior Correlation Analysis

After controlling for age, sex, and mean FD, a whole-brain correlation analysis of cue-triggered food-craving scores and fALFF was carried out. The threshold for significant regions was set at cluster *p* < 0.05, voxel *p* < 0.005, and two-tailed (GRF), which was implemented in DPABI software.

### 2.5. Region of Interest (ROI)-Wise FC Analysis

The coordinate of ROI seeds was selected according to the fALFF-behavior correlation results. We set each seed as a 6 mm-radius sphere in MNI stereotaxic space and then conducted a whole-brain functional connectivity analysis. Linear regression analyses were performed between cue-triggered food-craving scores, and z-maps of FC, age, sex, and head motion were controlled as the covariates. The analyses were conducted using SPM12 and DPABI. The threshold for significant regions was set at cluster *p* < 0.05, voxel *p* < 0.001, and two-tailed (GRF), and implemented in DPABI software.

### 2.6. Statistical Analyses

The behavioral level of analysis was conducted with IBM SPSS Statistics (v. 21). Firstly, we compared the error rate and reaction time of four trial types in both food and flower AX-CPT tasks. Next, the *d’*-context and *d’*-target indices were calculated, along with the difference between the groups. Subsequently, a mediation analysis was performed using PROCESS to evaluate whether cue-triggered food cravings and the corresponding brain regions can mediate the relationship between BMI or FMI (z score) and the reactive or proactive control. Bias-corrected bootstrap 95% confidence intervals were obtained with 5000 bootstrapped samples.

## 3. Result

### 3.1. Description and Comparison of Demographics

Note: BMI = body mass index; FMI = fat mass index; SMM = skeletal muscle mass. There was no difference in age or sex between the two groups. A significant difference was found in hunger degree (t(1,68) = 2.16, *p* = 0.035) and BMI (t(1,68) = 12.79, *p* < 0.00) between the two groups. There was also a significant difference in the z-score of FMI between the overweight and normal-weight group, t(1,68) = 7.10, *p* < 0.001. There was no difference in the SMM, t(1,68) = 2.67, *p* = 0.107 (see Table 1). These indices prove that our research effectively distinguished between the overweight and normal-weight group. In the following analyses, we controlled the age, sex, hunger degree, and SMM as covariates.

### 3.2. Group Comparison of d’-Context/Target Index in Food and Flower AX-CPT

**Figure 2:**
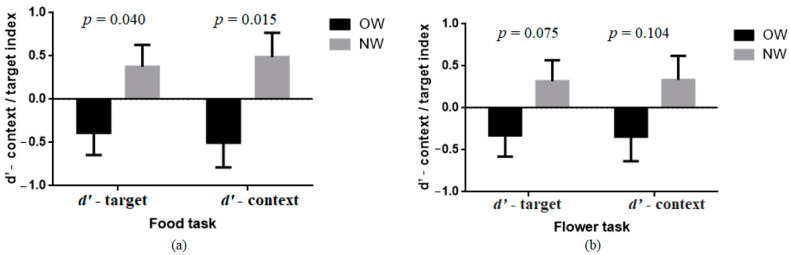
*d’*-context/target index in the AX-CPT as a function of BMI. Error bars represent within-subject standard errors of the mean; (**a**) represents the index of the food task; (**b**) represent the index of the flower task.

The *d’*-target index and *d’*- context index in the food and flower tasks were analyzed by MANOVA, and sex, age, and hunger were controlled as covariates. The results are shown in Table 2 and Figure 2. In the food-target task, we found a significant difference between the overweight and normal-weight group in reactive control (*d’*-target index, *F*(1,64) = 4.39, *p* = 0.040, *η^2^* = 0.06) and proactive control (*d’*-context index, *F*(1,64) = 6.26, *p* = 0.015, *η^2^* = 0.09). In the flower-target task, overweight people performed worse, but the difference did not reach a meaningful level. According to the signal-detection theory, the *d’*-context index indicates sensitivity to cue information. Hence, a higher value of the *d’*-context index represents better proactive control ability. Further, the *d’*-target index shows sensitivity to target information. Thus, a higher value of the *d’*-target index represents a better reactive control ability. Therefore, the results illustrate that only in food-target tasks, overweight people had significantly worse reactive control and proactive control ability than normal-weight individuals.

### 3.3. Mediation from FMI (z-Score) to Reactive and Proactive Control through Cue-Triggered Food Cravings

**Figure 3:**
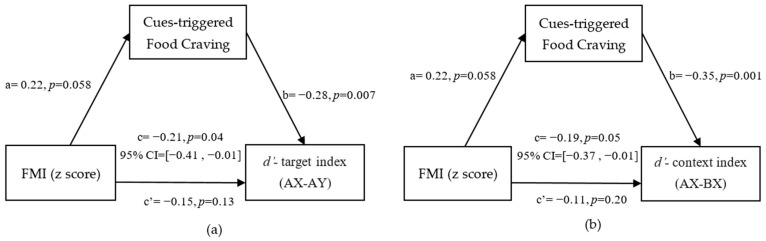
Mediation model and confidence intervals: Cue-triggered food cravings completely mediated the relationship between FMI (z-score) and the *d’*-target index (**a**) and *d’*-context index (**b**) after controlling for age, sex, and hunger degree. The standardized regression coefficients are shown in the path diagram. The significance level for corrections was set at *p* < 0.05.

The results confirmed our third hypothesis, that cue-triggered food cravings mediated the association of the FMI (z-score) with both the *d’*-target index and *d’*-context index. This result indicates that the negative associations of the FMI (z-score) with reactive control and proactive control were mediated by cue-triggered food cravings. In particular, cue-triggered food cravings could completely regulate the relationship between the FMI (z-score) and reactive control index (indirect: 95% CI = [−0.41, −0.01]; see Figure 3a).We also found that cue-triggered food cravings completely mediated the relationship between the FMI (z-score) and the proactive control index (indirect: 95% CI = [−0.19, −0.01]; see Figure 3b).

### 3.4. Mediation from BMI to Reactive and Proactive Control through Cue-Triggered Food Cravings

**Figure 4:**
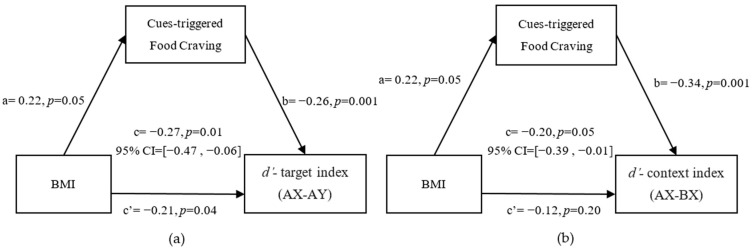
Mediation model and confidence intervals: Cue-triggered food cravings partially mediated the relationship between BMI and *d’*-target index (**a**), and completely mediated the relationship between BMI and the *d’*-context index (**b**) after controlling for age, sex, hunger degree, and FD. The standardized regression coefficients are shown in the path diagram. The significance level for corrections was set at *p* < 0.05.

The results confirm that cue-triggered food cravings mediated the association of BMI with both the *d’*-target index and *d’*-context index. This result indicates that the negative associations of BMI with reactive and proactive control were mediated by cue-triggered food cravings. In particular, cue-triggered food cravings partially mediated the relationship between the BMI and the reactive control index (indirect: [95% CI = −0.16, −0.01]; see Figure 4a).We also found that cue-triggered food cravings completely regulated the relationship between BMI and the proactive control index (indirect; [95% CI = −0.23, −0.01]; see Figure 4b).

### 3.5. Mediation from FMI (z-Score) to Reactive and Proactive Control through FC between Bilateral SFG_Orb

To explore whether the activation of brain regions related to cue-triggered food cravings is the underlying neural mechanism of FMI-influenced reactive control or proactive control, we first correlated cue-triggered food cravings with the fALFF of each brain voxel to obtain the exact brain regions. After adjusting for age, sex, and FD, cue-triggered food cravings were positively associated with the fALFF in the right superior frontal gyrus and orbital part (SFG_Orb_R; see Table 3). A whole-brain functional connection analysis was performed after setting the right SFG_Orb as ROI. We found that cue-triggered food cravings were positively associated with RSFC strength between the right SFG_Orb and bilateral SFG_Orb (see Table 3).

**Figure 5:**
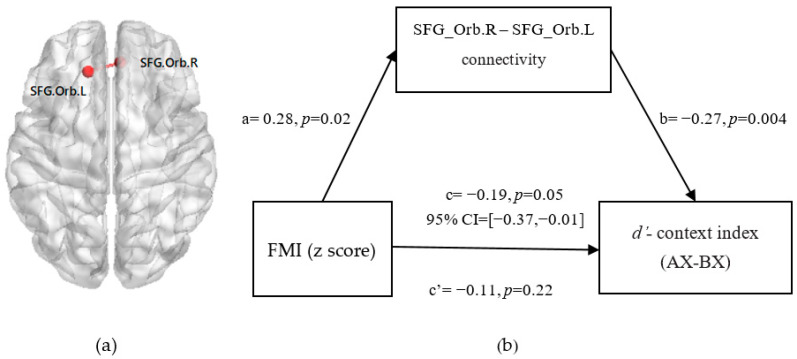
The FC between the bilateral SFG_Orb was significantly related to cue-triggered food cravings (**a**). Mediation model and confidence intervals: The FC between FMI (z score) and the *d’*-context index (food task) was fully mediated by the FC between the bilateral SFG_Orb after adjusting for age, sex, hunger degree, and FD (**b**). The standardized regression coefficients are presented in the path diagram. The significance level for corrections was set at *p* < 0.05.

Furthermore, we tested the mediation model of whether the FC between the bilateral SFG_Orb mediated the association of the FMI (z-score) with reactive control and proactive control (see Figure 5a), and set age, sex, and hunger degree as covariates. The results show that the FC between the bilateral SFG_Orb mediated the relationship between FMI (z-score) and proactive control (indirect 95% CI = [−0.37, −0.01]; Figure 5b). However, the path coefficient from FMI (z score) to reactive control was insignificant (indirect: 95% CI = [−0.09, 0.10]).

### 3.6. Mediation from BMI to Reactive and Proactive Control through FC between Bilateral SFG_Orb

**Figure 6:**
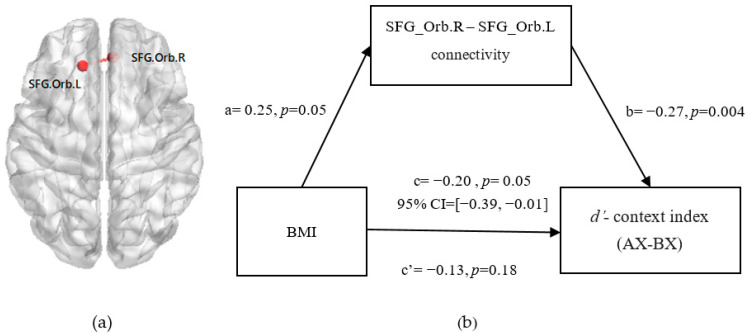
The FC between the bilateral SFG_Orb was significantly related to cue-triggered food cravings (**a**). Mediation model and confidence intervals: The FC between BMI and the *d’*-context index (food task) was fully mediated by the FC between the bilateral SFG_Orb after adjusting for age, sex, hunger degree, and FD (**b**). The standardized regression coefficients are presented in the path diagram. The significance level for corrections was set at *p* < 0.05.

Furthermore, we tested the mediation model of whether the FC between the bilateral SFG_Orb mediated the association of the BMI with reactive control and proactive control (see Figure 6a), and set age, sex, hunger degree, and MMV as covariates. The results show that the FC between the bilateral SFG_Orb mediated the relationship between BMI and proactive control (indirect 95% CI = [−0.22, −0.01]; Figure 6b). The path coefficient from BMI to reactive control was insignificant (indirect: 95% CI = [−0.12, 0.01]).

## 4. Discussion

Although previous studies have found that overweight people performed worse on food stimulation cognitive control tasks than general stimulation tasks [22,23], they still lack empirical research from the perspective of the cognitive process. Thus, we first applied the AX-CPT to overweight individuals to explore the impact of BMI on cognitive flexibility in the two control mechanisms. The proactive control process needs to suppress the previous task settings and activate the current rules. Compared to people of normal weight, overweight people performed significantly worse on food stimulation tasks and marginally significantly worse on general stimulation tasks. This result shows that being overweight negatively affects the proactive control process. This result validates the findings of previous studies that overweight or obese people have poor inhibition and control ability and are more likely to act impulsively [39,40,41]. Furthermore, a significant difference was found in the reactive control process, especially in food stimulation tasks, indicating that participants with excessive weight performed worse in this task. Considering that food has a more substantial rewarding effect on overweight individuals, they are more likely to be distracted during food stimulation tasks. Thus, they spend more cognitive resources to complete the tasks. This result verifies Hypothesis 1.

Further analysis revealed that cue-triggered food cravings partially mediated the relationship between BMI and reactive control, and completely mediated the relationship between BMI and proactive control in the food stimulation task. The results also show that cue-triggered food cravings completely mediated the relationship between FMI (z-score) and reactive and proactive control. Moreover, these results suggest that food cravings can accurately explain the relationship between overweight and the cognitive-switching process. This result verifies Hypothesis 2. Overweight individuals tended to have higher food cravings [42] and showed an approach-avoidance pattern of attention allocation toward high-energy food [43], resulting in impaired performance in the switching tasks with food stimulation. Food-related attentional bias is sensitive to changes in the motivational and rewarding value of food [44]. Their first gaze on food was related to cravings, but then showed reduced maintenance of attention to the food stimulation [43]. Event-related potential evidence also supports this view. In food-related cognitive switching tasks, overweight people might be less efficient in monitoring and resolving conflicts and require more cognitive resources to deal with food-related stimuli [22]. Based on the dual competition framework theory [45], we believe that the competition between cravings (emotion and motivation) and executive control leads to dynamic changes in the allocation of cognitive resources. Previous studies have shown that monetary rewards may lead to a significant decline in the cost of inhibition control and switching [46]. For this reason, overweight people should allocate more cognitive resources to inhibiting food cravings [22,42]. Overall, these findings suggest that the mediating effect of food cravings on the indicators of overweight and the switching process is related to the distribution of attention and cognitive resources. Consistent with previous opinions, we also found some differences in the underlying cognitive mechanisms of food cravings on proactive and reactive control. To maintain a continuous display of prompt information, proactive control requires more cognitive resources than reactive control [18]. Therefore, we inferred that proactive control is more likely to be adversely affected than reactive control when cognitive resources are limited. Hence, food cravings can completely mediate the relationship between overweight and proactive control. Meanwhile, food cravings mediated the relationship between overweight and reactive control to a certain extent. We speculate that other influencing factors should affect this relationship, which needs to be explored in the future.

We further discovered the similarity in the mechanism between food cravings and addiction. Based on resting-state fMRI imaging data, we found a positive correlation between food cravings and the functional connection between the bilateral OFC, which can completely mediate the relationship between BMI and proactive control. This result partially verifies Hypothesis 3. Previous neuroimaging studies have shown that overweight or obese participants exhibited abnormal activation in food-related reward tasks [29,30]. Similar to the results found in the food-tasting task, OFC activation indicates pleasantness [47,48]. Furthermore, OFC is considered a region related to addiction [49,50]. In the study on marijuana addiction, it was found that in the addict group, self-reported craving scores were positively correlated with brain activation in the ventral striatum and the medial and lateral OFC [51]. Similar underlying mechanisms have also been revealed between drug addiction and food addiction in obese individuals, especially in brain regions associated with rewards, including the ventral striatum and OFC [27,28]. Moreover, the OFC is not only involved in reward cravings but also reward monitoring and decision-making. Several studies on reward-evaluating tasks (including high-energy food cues) have found hyperactive activation in reward-related brain regions [52,53,54]. Previous studies demonstrated that in reward–decision tasks, enhanced connectivity between regions participating in reward evaluation could predict impaired cognitive control of overweight participants [55]. The results further prove that food cravings and OFC might be the key mechanisms of impaired cognitive flexibility in overweight individuals. However, we did not distinguish between the different neural mechanisms of reactive and proactive control.

Altogether, consistent with the different paradigms used in previous studies [22,23], we found that overweight people showed worse cognitive switching ability in food tasks. These results provide new evidence for the underlying mechanism of weaker cognitive control in overweight people. The previous study suggested that overweight people have greater food choice-related motivational and emotional responses, especially to high-calorie food [56,57]. Overweight people usually exhibited a preference for high-calorie foods but suboptimal nutrient intake (e.g., vitamins, dietary fiber) leads, in turn, to a higher risk of weight gain [56,58,59]. The imbalanced energy diet is one of the main causes of the increased prevalence of obesity [60]; lower diet nutritional quality was more likely to have a significant increase in BMI [61]. Therefore, it is important for overweight people to consider the healthiness of the food and inhibit the craving for high-energy foods. A six-week follow-up cognitive training for overweight people significantly reduced approach bias for unhealthy food and increased healthy food choices [62]. The result inspired us in further clinical studies. Cognitive intervention to reduce food cravings in overweight individuals can improve their ability to resist the temptation of high-energy foods and balance their nutrient intake. This may be a feasible strategy to help reduce body weight in the long term. Moreover, the present study found that food cravings and the FC between the bilateral OFC can mediate the relationship between BMI/FMI (z-score) and proactive/reactive control, respectively. Meanwhile, the functional connection between the bilateral OFC might be the target area in modulating overweight or obesity for several physiotherapies, such as transcranial electrical stimulation or transcranial magnetic stimulation. The results provide a new perspective for interventions in people suffering from obesity.

There were some limitations to the current study. First, although the present study provides novel evidence to prove that overweight impairs cognitive flexibility in the two stages of cognitive processes, horizontal experiments cannot show the causal relationship between overweight and cognitive flexibility. Therefore, longitudinal studies can be designed to explore causal relationships between variables. Second, we also tried to discover the different mechanisms of overweight in the two stages of cognitive processes. We found that the FC of the bilateral SFG_orb can mediate the relationship between overweight indicators and proactive control, but not reactive control. Thus, further analysis is needed to explore the neural mechanisms of the indicators of overweight on reactive control. Third, more rigorous and effective control of influencing factors is necessary. The influencing factors, such as hydration and physical activity, should be controlled in the follow-up research.

## 5. Conclusions

The study compared whether the effects of overweight on cognitive flexibility are different in the food-target task and the flower-target task. We also explored overweight impaired cognitive flexibility at different stages of the cognitive process. We found that overweight people performed worse in the food-target task in both control mechanisms through the AX-CPT task. Meanwhile, cue-triggered food cravings mediated the relationship between overweight and the two control indicators. Moreover, the FC between the bilateral orbital frontal lobes corresponding to cue-triggered food cravings also mediated the relationship between BMI, FMI (z-score), and proactive control. This result also shows that the influence of overweight on reactive control and proactive control is different. Nonetheless, the underlying mechanism that affects reactive control remains to be explored. Thus, these findings provide new evidence for studying the mechanism by which being overweight affects cognitive flexibility.

## Figures and Tables

**Table 1:** Demographic description and group difference of overweight and normal-weight participants (*N* = 69).

	Overweight Group (*n* = 34)		Normal-Weight Group (*n* = 35)			
	Mean	SD	Mean	SD	*t*	*p*
Age (years)	19.71	1.40	19.63	0.69	0.29	0.772
Sex (male%)	50%	-	37%	-	1.07	0.288
Hunger	66.82	23.62	54.51	23.78	2.16	0.035
BMI (kg/m^2^)	27.65	2.49	21.16	1.66	12.79	0.001
FMI (z score)	0.42	0.75	−0.88	0.76	7.10	0.001
SMM (kg)	22.98	4.38	20.66	4.98	2.67	0.107

**Table 2:** Group difference of *d’*-context/target index in the AX-CPT.

		OW (*n* = 34)	NW (*n* = 35)			
		M (SD)	M (SD)	*F*	*p*	*η^2^*
Food task	*d’*-target index	−0.39 (1.94)	0.38 (0.99)	4.39 *	0.040	0.06
	*d’*-context index	−0.50 (2.09)	0.49 (1.00)	6.26 *	0.015	0.09
Flower task	*d’*-target index	−0.33 (1.82)	0.32 (1.10)	3.27	0.075	0.05
	*d’*-context index	−0.34 (2.20)	0.33 (1.05)	2.72	0.104	0.04

Note: AX-CPT = AX-continuous performance test; OW = overweight; NW = normal weight; * *p* < 0.05.

**Table 3:** Regions where fALFF and RSFCs were significantly associated with cue-triggered food cravings.

Regions	Side	BA	Peak MNI Coordinates			Peak Intensity	Cluster Size (Voxels)
			X	Y	Z		
Correlation with fALFF							
Superior frontal Gyrus_Orb	R	11	6	42	−27	4.105	24
SFG_Orb as the seed							
Superior frontal Gyrus_Orb	L	11	−15	36	−24	5.359	55
Superior frontal Gyrus_Orb	R	11	12	47	−18	6.374	75

Notes. The threshold for significant regions was set at cluster *p* < 0.05, voxel *p* < 0.001, two tailed (GRF). SFG_Orb = superior frontal gyrus, orbital part; L = left; R = right; BA = Brodmann area; MNI = Montreal Neurological Institute.

## Data Availability

The data that support the findings of this study are available from the corresponding author upon reasonable request.
